# Detection of a Novel Phlebovirus (Drin Virus) from Sand Flies in Albania

**DOI:** 10.3390/v11050469

**Published:** 2019-05-23

**Authors:** Silvia Bino, Enkelejda Velo, Përparim Kadriaj, Majlinda Kota, Gregory Moureau, Xavier de Lamballerie, Ani Bagramian, Remi N. Charrel, Nazli Ayhan

**Affiliations:** 1 Institute of Public Health, Tirana 1001, Albania; silviabino@gmail.com (S.B.); keladikolli@yahoo.com (E.V.); pkadriaj@yahoo.com (P.K.); mdhimolea@live.com (M.K.); 2 Unite des Virus Emergents (Aix-Marseille Univ–IRD 190–Inserm 1207–IHU Mediterranee Infection), 13005 Marseille, France; gregory.moureau@univ-amu.fr (G.M.); xavier.de-lamballerie@univ-amu.fr (X.d.L.); ani.bagramian@yahoo.fr (A.B.); remi.charrel@univ-amu.fr (R.N.C.); 3 Emerging Pathogens Institute, University of Florida, Gainesville, FL 32608, USA

**Keywords:** phlebovirus, sand fly-borne phleboviruses, sand fly fever, sand fly fever Sicilian (SFSV) virus, Albania

## Abstract

Phlebotomine sand flies are generalist vectors with significant implications for public health. They are able to transmit phleboviruses that cause sand fly fever, headaches, or meningitis in humans. Albania is a country in Southeast Europe with a typical Mediterranean climate which provides convenient conditions for the presence of sand flies. Hence, the circulation of phleboviruses, such as the Toscana and Balkan viruses, has been recently described in the country. We followed a virus discovery approach on sand fly samples collected in 2015 and 2016 in seven regions of Albania, with the aim to investigate and characterize potentially circulating phleboviruses in phlebotomine sand flies. A presumed novel phlebovirus was detected in a pool consisting of 24 *Phlebotomus neglectus* males. The virus was provisionally named the *Drin virus* after a river near the locality of Kukës, where the infected sand flies were trapped. Genetic and phylogenetic analysis revealed that the Drin virus is closely related to the Corfou (CFUV) virus, isolated in the 1980s from *Phlebotomus major* sand flies on the eponymous island of Greece, and may also be involved in human infections because of its similarity to the sand fly fever Sicilian virus. The latter justifies further studies to specifically address this concern. Together with recent findings, this study confirms that Albania and the Balkan peninsula are hot spots for phleboviruses.

## 1. Introduction

According to their antigenic relationships, old world sand fly-borne phleboviruses (genus *Phlebovirus*, family *Phenuiviridae*, order *Bunyavirales*) can be classified into three serological complexes, which are also regarded as taxonomic species or tentative species—the *Sandfly fever Naples phlebovirus* species (SFNV), the *Salehabad phlebovirus* species, and the *Sandfly fever Sicilian phlebovirus* tentative species (SFSV). The SFNV includes the following viruses: Arrábida (ARRV), Balkan (BALKV), Fermo (FERV), Gordil (GORV), Granada (GRAV), Massilia (MASV), Punique (PUNV), Saddaguia (SADV), Saint-Floris (SAFV), sand fly fever Naples (SFNV), Tehran (THEV), Toscana (TOSV), and Zerdali (ZERV) (https://talk.ictvonline.org/taxonomy/). The *Salehabad phlebovirus* species includes Adana (ADAV), Adria (ADRV), Alcube (ALCV), Arbia (ARBV), Arumowot (AMTV), Medjerda Valley (MVV), Odrenisrou (ODRV), Olbia (OLBV), Salehabad (SALV), Bregalaka (BREV), and Zaba (ZABAV) (https://talk.ictvonline.org/taxonomy/). The SFSV tentative phlebovirus species is a serocomplex which includes several phlebovirus species that are yet to be recognized by the International Committee on Taxonomy of Viruses (ICTV). The SFSV and Corfou (CFUV) phlebovirus tentative species are antigenically and genetically close to each other and may be grouped together as a unique species [1,2,3].

Most phleboviruses use sand flies as vectors [4]. Phlebovirus distribution and circulation peaks are closely related to vector presence and activity. Sand flies are broadly distributed in regions bordering the Mediterranean basin. They are abundant in peri-urban and rural environments, which are concentrated near human and domestic animal habitats [5]. Therefore, human populations are exposed to sand fly–transmitted diseases, among them phlebovirus-related diseases. For example, TOSV, which was isolated in Italy in 1971, is of major concern as it causes central nervous system infection leading to acute meningitis in humans, whereas the SFNV and SFSV only induce a transient febrile illness known as the “three-day fever”, “phlebotomus fever”, or “pappataci fever” [6,7,8]. The ADRV, detected in phlebotomine sand flies in Albania, is the only virus that is found to be associated with human disease within the *Salehabad phlebovirus* species [9,10]. Phleboviruses are emerging pathogens that raise veterinary and public health concerns. Recently, a large number of novel phleboviruses have been identified in the Mediterranean area and have been included in existing species or in the tentative ones detailed above.

Albania has a typical Mediterranean climate that provides convenient environmental conditions for the active circulation of sand flies between May and November. To date, a total of eight sand fly species have been described in Albania, *P. neglectus* being the most prevalent (75.6%), followed by *P. perfiliewi* (14.4%), *P. papatasi* (4.6%), *P. tobbi* (3.6%), *P. similis* (1.8%), *P. simici* (<1%), *Sergentomyia dentata* (<1%), and *Sergentomyia minuta* (<1%) [11,12].

Recently, several phleboviruses were identified in specific areas of Albania—the BALKV virus, belonging to the SFNV species and the ADRV virus, belonging to the *Salehabad phlebovirus* species [9,13].

Our aim was to investigate and characterize the phleboviruses in phlebotomine sand flies collected in more than one region in Albania from June to September of 2015 and 2016.

## 2. Material and Methods

### 2.1. Sand Fly Trapping

**Figure 1:**
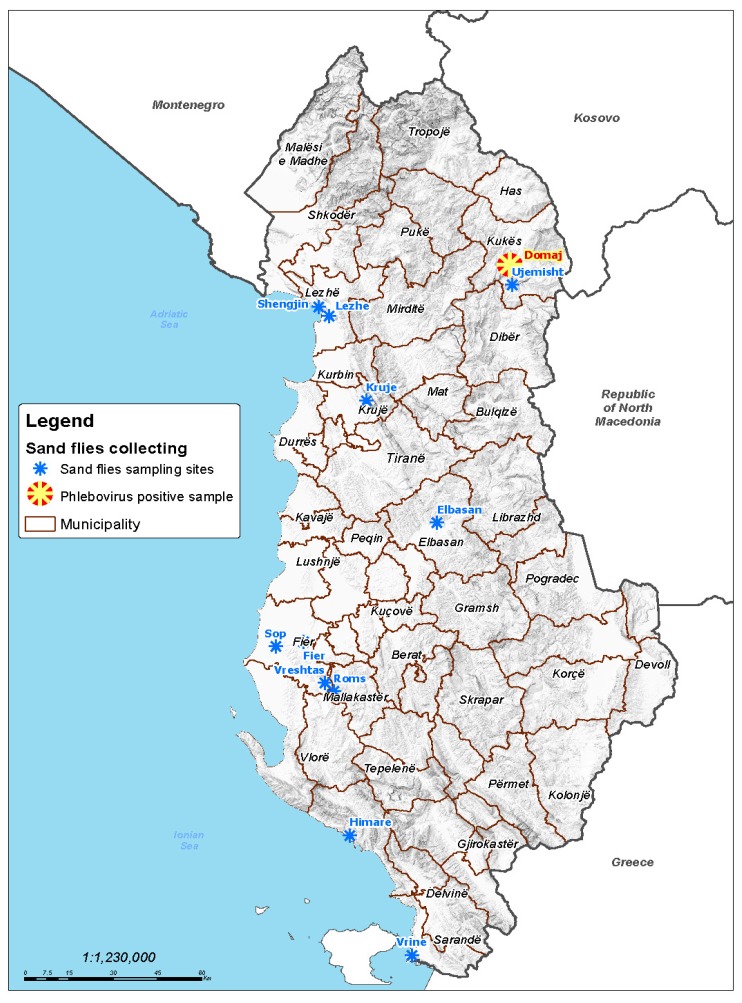
Sand fly sampling regions and phlebovirus positive pool location.

CDC miniature light traps and CO_2_ baited traps (BioQuip Products, Rancho Dominguez, CA, USA) were used to collect sand flies in seven regions of Albania from June to September of 2015 and 2016 (Figure 1).

Live sand flies were placed on a solid–CO_2_ container for transportation to the laboratory and pooled in 1.5 mL tubes based on sex, trapping site, and trapping day, with up to 30 individuals per pool. The tubes were stored at −80 °C. No morphological identification of the sand flies was performed prior to viral testing to avoid virus or RNA degradation [13,14].

### 2.2. Sand Fly Homogenization, Tentative Isolation in Ncell Lines and Baby Mice

A volume of 600 μL of Eagle’s minimal essential medium (EMEM) (enriched with 5% fetal bovine serum (FBS), 1% penicillin–streptomycin, 1% (200 mM) l-glutamine, 1% kanamycin, and 3% amphotericin B (Fungizone)) with 3 mm tungsten beads added into tubes. Sand fly tissues were homogenized by a Mixer Mill MM300 (Qiagen, Courtaboeuf, France), and the mixture was centrifuged at 5800× *g* for 10 min.

A volume of 50 μL homogenate supernatant was inoculated into 450 μL Vero cells in enriched EMEM without FBS. The cells were then incubated at room temperature for 1 h. After 1 h, 2.5 mL of fresh EMEM with 5% FBS was added and the nucleons incubated at 37 °C in a 5% CO_2_ atmosphere. The nucleons were examined daily for the presence of a cytopathic effect (CPE) and passaged 8 times. In each passage, 200 μL of supernatant medium was collected from the nucleons and tested by reverse transcription PCR (RT-PCR) with stratifin (SFN), Nphlebo, and Lambert primers [14,15,16,17].

A volume of 2 μL of the original homogenate supernatant was used in an attempt to infect 2 to 3 day-old Oncins France 1 (OF1) mice intracerebrally. The mice were observed for signs of disease and sacrificed on days 6 to 14 of the experiment. These animal experiments were carried out according to the animal experiment regulations of Aix-Marseille Université under the license A1301309. The RNA was extracted from the brain tissues of the mice by using a QIAcube HT system with a QIAamp 96 Virus QIAcube HT Kit (Qiagen, Hilden, Germany) according to the manufacturer’s instructions and studied for the presence of the Drin virus.

### 2.3. Nucleic Acid Extraction and Molecular Assays

A volume of 200 μL homogenate supernatant was used for nucleic acid extraction. For the 2015 sand fly samples, the extraction was performed by a BioRobot EZ1-XL Advanced (Qiagen, Hilden, Germany) with a Virus Extraction Mini Kit (Qiagen). The samples from 2016 were extracted using QIAcube HT system with a QIAamp 96 Virus QIAcube HT Kit (Qiagen, Hilden, Germany).

RT-PCR was performed with the Super Script III (Thermo Fisher Scientific) kit using three different systems: (1) the Nphlebo1S/1R system targeting the polymerase gene in the L RNA segment, (2) the SFNV1S/1R system targeting the nucleoprotein gene in the S RNA segment, and (3) the Lambert Phlebovirus system, also targeting the S RNA segment [14,15,16,17]. The cycling program of the RT-PCR reaction consisted of 48 °C for 45 min and 94 °C for 2 min, followed by 40 cycles at 94 °C for 30 s, the annealing temperature for 1 min, and 68 °C for 45 s, with a final elongation step at 68 °C for 7 min.

Nested PCRs were performed with a DreamTaq kit (Thermo Fisher Scientific, Waltham, MA, USA) with Nphlebo2 and SFNV2 primers. The following cycle was used for the Nested-PCRs: 94 °C for 5 min, followed by 40 cycles at 94 °C for 30 s, the annealing temperature for 45 s, and 72 °C for 30 s, with a final elongation step at 72 °C for 7 min.

Real-time quantitative RT-PCR (RT–qPCR) was performed with the EXPRESS qPCR SuperMix Universal (Thermo Fisher Scientific, Waltham, MA, USA) kit and the Quant Studio 12K Flex System. The cycling program of the RT-qPCR reaction consisted of 50 °C for 5 min and 94 °C for 20 min, followed by 40 cycles at 95 °C for 3 s, then 60 °C for 30 s. Specific primers were used to screen for the SFSV group viruses and TOSV [8,18].

The Drin virus was identified as closely related to the Corfou virus based on the partial sequence obtained from the Nphlebo primers in the polymerase (L RNA).

**Figure 2:**
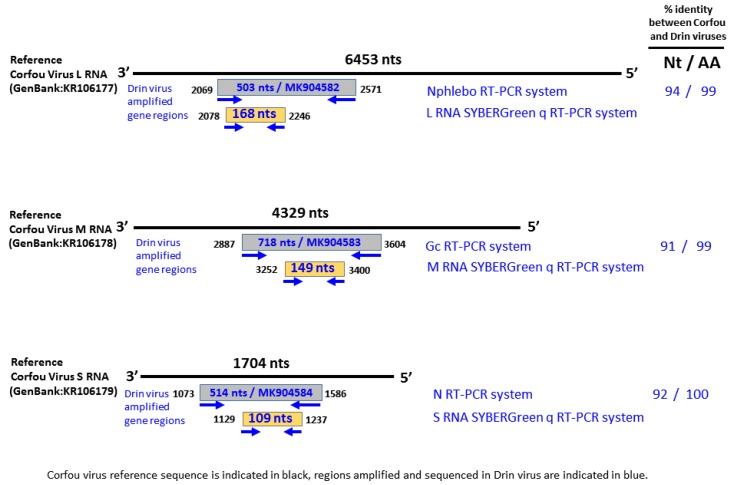
Schematic representation of specifically designed primers.

Because of the closeness between the polymerase partial sequences of the Drin and CFUV viruses, we designed new primers using the CFUV virus sequences as a reference. We assumed that these primers would also amplify the Drin virus segments (Table 1; Figure 2).

Additionally, we designed real-time RT-qPCR assays specifically targeting the Drin virus L-, M- and S-segments to test all the pools to check whether the Drin virus sequences were present in any of the other analyzed pools (Table 1; Figure 2). The same protocols were used for RT-PCR and real-time RT-qPCR, as described above.

The positive PCR products were purified using a QIAquick PCR purification kit (Qiagen, Hilden, Germany) and sequenced with next-generation sequencing (NGS) using an AU4c Ion-Torrent PGM platform as described [19].

### 2.4. Phylogenetic Analysis

The sequence data was analyzed through CLC Genomics Workbench version 11 software (Qiagen, Hilden, Germany). Homologous sequences from phleboviruses were aligned with homologous sequences of interest using Clustal W in Molecular Evolutionary Genetics Analysis 6 (MEGA6) software [20]. The evolutionary history was inferred by the neighbor–joining (NJ) method using the Kimura 2-parameter model with 1000 bootstrap replication.

### 2.5. Genotyping the Sand Flies of Virus-Positive Pool

To identify the sand fly species constitutive of the Drin virus positive pool, barcoding PCR was performed with 3 μL of nucleic acid to amplify partial regions of the cytochrome *c* oxidase I (*COI*) and cytochrome *b* (*Cytb*) genes, respectively, as previously described [21,22]. The PCR products were purified and sequenced through NGS. The reads were compared with available sequences in the GenBank sequence database using CLC Genomic Workbench 11 software (Qiagen, Hilden, Germany).

## 3. Results

### 3.1. Sand Fly Collection and Virus Testing

A total of 2502 sand flies were tested for phlebovirus presence, 533 collected in 2015 (298 females and 244 males), and 1969 in 2016 (1064 females and 905 males) in seven different regions of Albania (Figure 1). They were organized in a total of 118 pools (27 and 91 pools in 2015 and 2016, respectively) which were tested as described above. Pool #17, containing 24 male sand flies collected in 2015 in the village of Domaj, in the municipality of Kukës, provided a PCR product of the expected size with polymerase Nphlebo primers [16], and a positive result with SFSV RT-qPCR tested using SFSV primers (Ct = 26,9) that are specific for SFSV and CFUV viruses [8]. The real-time RT-qPCR assay designed from CFUV L, Gc, and S RNA sequences was positive with Ct values <29. Of the 118 pools that were studied, only Pool #17 was found to be positive. Genotyping the sand flies of the virus-positive Pool #17 by NGS analysis of the PCR product provided 6910 reads for the *COI* gene and 397 reads for the *Cytb* gene. All 7307 reads 100% matched the *P. neglectus* sequences, and thus demonstrated that the 24 sand flies contained in Pool #17 belonged to the *P. neglectus* species.

### 3.2. Attempts of Virus Isolation

Vero cells inoculated with supernatant corresponding to Pool #17 were examined daily for CPE. Eight blind passages were done, but no CPE was observed. In addition, each passage was tested for the presence of Drin virus RNA using the SFN, Nphlebo, and Lambert sets of primers; the results were consistently negative, indicating that the Drin virus was not isolated in cell culture. For this reason, we decided to try virus isolation by using intracerebral (IC) inoculation into suckling mice. A total of 25 baby mice were injected with sand fly homogenate supernatant with the attempt to isolate the Drin virus. None of the mice developed clinical signs, which suggested that the Drin virus was not replicating in their brain tissues. Baby mice were euthanized at 7 days post-IC injection, the brain was then collected postmortem, with nucleic acid extraction performed as described above and tested with different PCR assays. All results were negative, showing that the Drin virus had not been isolated.

### 3.3. Genetic Characterization and Phylogenetic Analysis of Drin Virus

Partial sequences of the three RNA segments (GenBank accession numbers: MK904582, MK904583, and MK904584) were amplified and sequenced. In all cases, the Drin virus sequences were most closely related to, but clearly distinct from, those of the CFUV virus (Figure 2).

**Figure 3:**
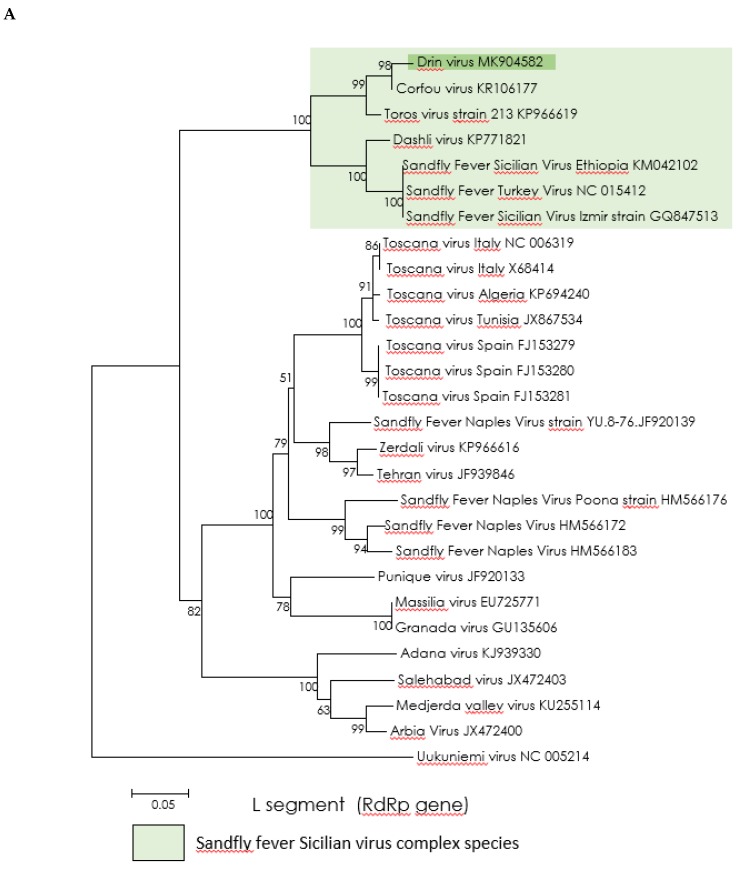

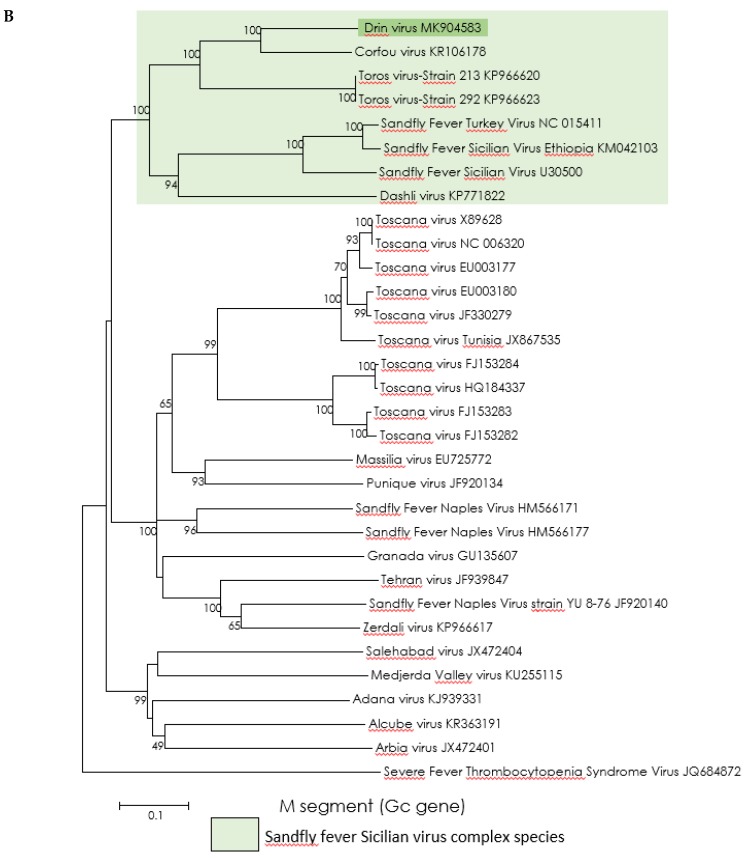

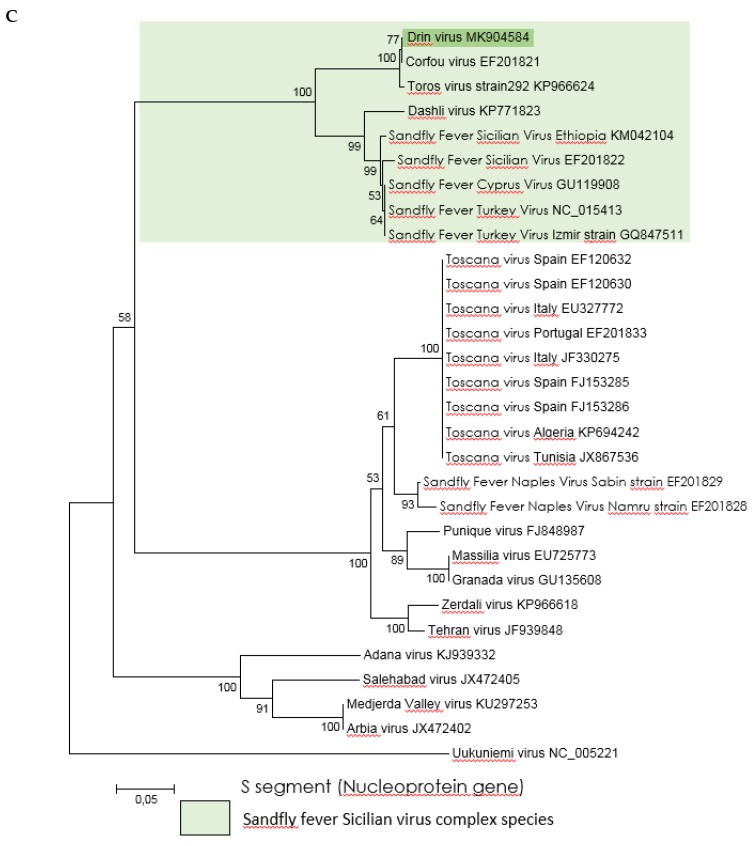
Phylogenetic analysis of the phlebovirus amino acid sequence. Neighbor-joining (NJ) phylogeny trees were generated using the Kimura 2-parameter model with 1000 bootstrap replication. (**A**) L protein amino acid sequence (167 AA). (**B**) Gc protein amino acid sequence (170 AA). (**C**) Nucleoprotein amino acid sequence (170 AA). GenBank accession numbers are inserted in the taxon’s nomination.

Regardless of the gene used for analysis, the Drin virus is grouped with the CFUV and Toros viruses (with 99–100% bootstrap support), the three viruses forming a sister cluster to the group including the Dashli virus and strains of SFSV (Figure 3).

All these viruses form a solid monophyletic cluster which is consistent with the proposal that they should all be placed within a single species, one that is yet to be recognized by the ICTV as a SFSV species.

## 4. Discussion

The viruses belonging to the SFSV and CFUV tentative phlebovirus species have previously been described in Italy, Turkey, Cyprus, Greece, Iran, and Ethiopia, but they have never been detected in Albania or the other countries of the Western region of the Balkans [1,3,23,24,25,26]. Although SFSV and CFUV viruses were respectively discovered in 1943 and 1985, both remained classified as tentative species within the genus *Phlebovirus*. Recently, the isolation of the Dashli virus in Iran has been an opportunity to propose to group SFSV, CFUV, and Dashli viruses within a single species putatively named *Sandfly fever Sicilian phlebovirus species* [3].

In this study we identified a novel phlebovirus, tentatively named the Drin virus after the eponymous river in the Kukës region of Albania. The virus was identified in a pool containing only male sand flies belonging to the *P. neglectus* species which is the predominant species in that area. Other studies have demonstrated that phleboviruses were potentially transmitted by several distinct species of sand flies [27]. The second interesting finding is that the pool containing Drin virus RNA consisted exclusively of male sand flies—this has been previously reported in most of the studies on phlebovirus detection in sand flies [19,28,29,30], and is related to trans-ovarial or venereal transmission [31]. Assuming that only one individual was infected in Pool #17, the sand fly infection rate was 0.18% during 2015 and 0.04% if both years are combined. For comparison, infection rates were 0.04% for the Dashli virus in Iran and 0.026% for the Toros virus in Turkey [3,32]. There is no data to allow us to calculate the infection rate for the CFUV virus in Greece [1]. Sand fly trapping sites were scattered to best cover the whole country (Figure 1). As previously reported [32], NGS analysis of PCR products within the *COI* and *Cytb* genes of sand flies is an efficient approach for species identification within a pool of insects. Here, all sand flies included in Pool #17 were *P. neglectus*, suggesting that this species is capable of transmitting the Drin virus between sand flies and possible vertebrate hosts. As *P. neglectus* is widespread in the Balkan peninsula, in Eastern Europe, and in Turkey [5], and because it was involved in the transmission of the Balkan virus [13] and Toscana virus [33], competence studies are needed to understand better the potential of this species to transmit viruses of medical importance.

Gene by gene comparative distance analysis showed that pairwise distances of the Drin virus versus CFUV and Toros viruses were consistently lower than the lowest distances observed between Drin virus and SFSV–Dashli virus. Detailed genetic distance analyses have been done for the SFSV and CFUV phlebovirus tentative species by Alkan et al. [3].

The limited genetic divergence between partial sequences of the Drin and CFUV viruses questions whether they are two distinct viruses or two strains of the same virus. Since taxonomy does not regulate delineation below the species level, there may be contradictory elements. In favor of considering that the Drin and CFUV viruses are two strains of the same virus—they are genetically very close, and they have been detected in two species of sand flies belonging to the same subgenus (*Larroussius*). Evidence pointing to the Drin and CFUV viruses as two distinct viruses—they have been discovered in different species of sand flies, they were discovered in different countries, and the Drin virus was found in sand flies trapped in the northern part of Albania which has no contact with Corfou island that faces the Ionian coasts of Albania. It is not known whether cross-neutralization exists for the Drin and CFUV viruses, and the isolation of the Drin virus is required to conduct the proper experiments to address this question.

Therefore, we propose to maintain that the Drin virus and CFUV virus are two distinct representatives and look forward to further studies allowing the isolation of the Drin virus.

Overall, the aforementioned viruses are different members of the same species that remain unrecognized by the ICTV; currently, a proposal has been filled and submitted.

Interestingly, since the CFUV virus discovery 30 years ago, this is the first direct evidence that a virus belonging to the SFSV group is circulating in South East Europe. At the outset of this study, there was indirect evidence (neutralization-based seroprevalence studies) showing that SFSV was circulating in Portugal, but there is not any seroprevalence survey to document this in Albania [8].

As a consequence, there are representatives of the three main species of sand fly-borne phleboviruses in Albania (Balkan, Adria, and Drin viruses) and in the Balkan as well: Toscana and Balkan viruses for the *Sandfly fever Naples phlebovirus species*, Adria, Zaba and Bregalaka viruses for the *Salehabad phlebovirus species*, CFUV and Drin viruses for the tentative SFSV phlebovirus species.

SFSV has been described in an extended geographic area from southwestern Europe to the Middle East including northern Africa. SFSV epidemics were recorded in Italy, Cyprus, Turkey, and Ethiopia [23,27]. Furthermore, seroprevalence studies have shown the presence of neutralizing antibodies in humans in France and Cyprus [25,34]. In Iraq, during an outbreak among US Army troops in 2007, IgM antibodies specific for SFSV were detected in convalescent sera [35]. Specific IgG was also detected in Marine soldiers after self-reported febrile illness cases. A variant strain of SFSV was also isolated from human serum during an important outbreak in Ethiopia [26]. Although the pathogenicity of SFSV is not disputed [23,34], so far there are no data to support that the CFUV virus or genetic variants such as the Drin virus can cause disease in humans. The areas of circulation of viruses belonging to the SFSV phlebovirus species are wide enough to justify that efforts must be made to investigate the potential role of these viruses in human infections. Further seroprevalence studies may show indirect evidence of the circulation of the virus in humans. The fact that a single real-time RT–qPCR assay is capable of detecting all viruses that are so far recognized as members of this group is an important asset for studies of clinical cohorts of febrile illness in countries where direct or indirect evidence of the presence of these viruses have been reported.

## Figures and Tables

**Table 1:** Corfou (CFUV) virus specific primers.

Target	Assay	Name	Sequence (5′–3′)	Amplicon Size (bp)
L segment (*RdRp* gene)	real-time RT-qPCR	L-Corfou-Albania—Forward	AGCCACATAAGATGTGCAAG	168
		L-Corfou-Albania—Reverse	CCTGTGAAGGGATTGAACAT	
M segment (*Gc* gene)	RT-PCR	Gc—Forward	GAAGGACAACTGCTTAGCATG	718
		Gc—Reverse	CATTACAGGAATAACAGCCTG	
	Nested PCR	Gc-Nested—Forward	ATGCGGATGCTTCAATGTT	428
		Gc-Nested—Reverse	CATTCTATGATGTCAGTCAT	
	real-time RT-qPCR	Gc-RealTime—Forward	TTAGGTCTTCATCTGGAGC	149
		Gc-RealTime—Reverse	CATTCTATGATGTCAGTCAT	
S segment (*N* gene)	RT-PCR	N—Forward	TAGATGAGACCGTGGTCCA	514
		N—Reverse	GTTGATGGCGGCAGACAT	
	Nested PCR	N-Nested—Forward	ATGCCAAGAAGATGATTATTC	352
		N-Nested—Reverse	GTGAGCATCAACAATGGCAT	
	real-time RT-qPCR	N-RealTime—Forward	ATGGATAGAATCAGTGGTCAG	109
		N-RealTime—Reverse	GCTCCTCTGAGTCCAACAT	
